# Design of Key Technologies for Elderly Public Network Services Based on Intelligent Recommendations

**DOI:** 10.1155/2022/4592468

**Published:** 2022-10-04

**Authors:** Xinjia Zhang

**Affiliations:** School of Design Art, Changsha University of Technology, Hunan, Changsha 410114, China

## Abstract

As the world's population continues to increase, the proportion of elderly people is also rising. The existing elderly public service system is no longer able to meet the needs of the elderly for their daily lives. The elderly population is significantly less receptive to emerging matters than the younger population, resulting in the public elderly service system not being able to access the initial data of elderly users in a timely manner, which causes the system to make incorrect recommendations. Therefore, the elderly cannot enjoy all kinds of online services provided by the Internet platform. In order to solve this problem, an elderly intelligent recommendation method based on hybrid collaborative filtering is proposed. First, the data of elderly users and elderly service items are scored, and modelling is completed by a collaborative filtering algorithm. Then, the XGBoost model is combined to solve the optimal objective function, so that the recommended data set with the highest score in the nearest neighbour set is obtained. The experimental results show that the proposed hybrid algorithm effectively solves the cold start phenomenon that occurs when the elderly population uses the web to make recommendations for elderly services. In addition, the proposed hybrid algorithm has a higher recommendation footprint accuracy than other recommendation algorithms.

## 1. Introduction

As living standards continue to rise, people are becoming more and more concerned about health issues. At the same time, the medical system of the whole society is also improving, which has led to a trend of increasing human life expectancy. However, with the increase in the average life expectancy of the population, the issue of ageing has also received more attention [1–6]. With the gradual arrival of the aging population, the demand for public services of the middle-aged and elderly people in society is also increasing. However, in the context of population ageing, the existing social public elderly service system cannot effectively improve the living standard of the elderly, which is caused by various factors such as economic cost, human cost, and medical system [7–9]. Therefore, how to use advanced technology to improve the efficiency of the public elderly service system has become a key issue for research in related fields.

With the rapid development of the Internet, information technology has become a mainstream technical means of society. The existing social public elderly service system is still in its infancy in terms of information technology application. Most regions still rely on traditional management methods for their elderly services [10–13]. With the increasing knowledge level of the elderly, more and more of them are using mobile phones for online shopping or entertainment. Therefore, the trend of informatization of the social public elderly service system is also inevitable. The research direction of information-based elderly services generally refers to virtual community service technology. This technology usually involves the establishment of a unified information management service platform on a community basis. The corresponding elderly services are provided to the elderly through multiple platforms on the Internet. Virtual community service technology has two main key technologies [14, 15]: (1) interaction technology and (2) data management technology.

For elderly information, interaction technology is mainly used for communication between service staff and elderly users. Kang et al. [16] designed a service system based on Android OS and deployed it to a tablet terminal as well as a WEB platform. However, the system's service process is complicated when elderly users are operating on the web, thus greatly increasing the workload situation of service personnel. Sandelowski [17] has designed a virtual elderly service system that completes the service process by dialing a phone. The final payment is handled through a POS terminal. However, communication by telephone significantly increases the cost of the service.

For elderly information, data management techniques fall into two main categories: data mining systems and recommendation systems. Data mining systems are designed to monitor the daily behaviour of elderly people and store various aspects of data in a special database. Recommender systems make timely medical diagnostic recommendations based on data about the health status of elderly people or recommend certain activities based on their interests. For example, Castilla et al. [18] have designed a WEB-based predictive scoring system that can recommend an appropriate healthy diet based on an elderly person's daily activity level and daily mood. This system is well suited to the practical needs of elderly people. A recommendation system is a special form of information filtering system that determines a user's actual needs based on their preferences, thus enabling the provision of recommendations for them.

Research on recommendation systems began in the early 1990s. Recommendation algorithms are an important part of recommendation systems. According to the different recommendation strategies, recommendation algorithms are generally classified into content-based recommendation algorithms, collaborative filtering recommendation algorithms, and hybrid recommendation algorithms. Content-based recommendation algorithms have been applied in different Internet domains. Mishra et al. [19] proposed an intelligent agent system for web page recommendation. This intelligent recommendation is based on the user's evaluation of the web page to achieve intelligent recommendation. Deldjoo et al. [20] implemented a content-based recommendation system. This system can locate recommendations based on the user's evaluation information and the user's search statements. In contrast to content-based recommendation algorithms, collaborative filtering recommendation algorithms focus more on similar hobby recommendations for similar users. Collaborative filtering recommendation algorithm is suitable for systems with abundant users. Amazon was one of the first e-commerce sites to use a recommendation system, and it also uses collaborative filtering. In addition, hybrid recommendation algorithms combine multiple recommendation algorithms to take advantage of multiple strengths to achieve better recommendations.

Today, people can browse through a large number of real-time information and media resources via the Internet, and this is also true in the case of public elderly care service systems. For public aged care service systems, intelligent recommendation technology enables users to effectively save time in searching for resources on the platform, by improving the efficiency of their access to the Internet platform. Currently, this user intelligent recommendation technology has been effectively applied on many platforms [21–23].

However, with the rapid development of the Internet and big data technology, the public elderly service system has become more and more functional, resulting in an increasing amount of information on the Internet service platform. Sometimes, elderly users cannot find the part of the information they really need in the complex information, thus forming a typical information overload problem. Research has shown that existing recommendation systems must meet the prerequisite of high user activity. However, for elderly users, it is difficult to achieve accurate recommendations. A serious problem for this group of users is the low level of activity, which means that elderly users have relatively few reviews. This is because elderly users are less able to adapt to new things, resulting in the system being unable to produce accurate recommendations. The essence of this problem is the cold start problem of recommendations. The elderly population is significantly less receptive to new and emerging matters than the younger population, resulting in the public elderly service system not being able to access the initial data of elderly users in a timely manner, which causes the system to make incorrect recommendations.

Therefore, the aim of this study is to use a hybrid collaborative filtering algorithm to solve the recommendation cold-start problem for elderly users in a public elderly service system, so as to achieve personalised recommendation results that meet users' needs. There are currently more studies using collaborative filtering algorithms to complete user personalised recommendation services [24, 25]. Park et al. [26] applied collaborative filtering algorithms to a travel recommendation system to provide effective travel paths and tourist attractions based on user preferences. Zhuang [27] used the XGBoost model for Internet customer churn prediction analysis. Both algorithms have shown certain advantages in predictive recommendation systems. Therefore, this paper attempts to mix the two algorithms, to accomplish personalised recommendations for elderly users in public elderly service systems.

The main innovations and contributions of this paper include the followings:

1. Introducing collaborative filtering algorithms in intelligent recommendation technology into the field of public elderly service research, so as to complete personalised service recommendations in public elderly service systems
2. In order to solve the cold start problem and the sparsity problem of the elderly users' recommendation, this paper combines the XGBoost model and collaborative filtering algorithm and solves the optimal objective function through the weighted parameters, which solves the pension service recommendation for a special group of elderly people

The rest of the paper is organized as follows: in Section 2, the collaborative filtering recommendation model for elderly users was studied in detail, while Section 3 provides the proposed hybrid collaborative filtering algorithm.  Section 4 provides experimental results and analysis. Finally, the paper is concluded in Section 5.

## 2. A Collaborative Filtering Recommendation Model for Elderly Users

### 2.1. Public Elderly Care Service System for Elderly Users

The public elderly services system is a professional platform that provides online services for users. The system brings together the information of a number of businesses that provide services to elderly users. The system allows for the selection of the nearest service provider to the elderly user through the real-time location. In addition, a key feature of the system is the service recommendations based on the interests of the elderly.

**Figure 1:**
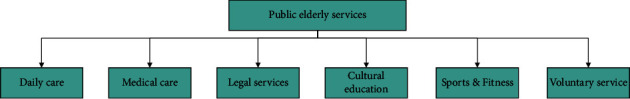
Functions of the public elderly care system.

The services provided by the public elderly service system mainly include daily care, medical care, legal services, cultural education, sports & fitness, and voluntary services, as shown in Figure 1. Detailed descriptions of the specific services are shown in Table 1.

**Figure 2:**
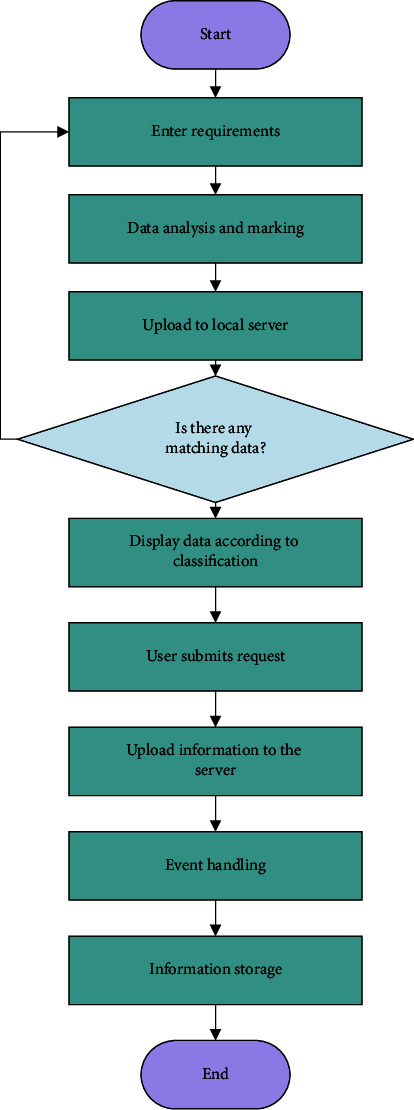
Flow of public elderly care services.

When an elderly user submits a personal request at the front end of the system, the system will calculate the merchant with the shortest path among all eligible merchants based on the user's current location. Finally, the system will also incorporate an intelligent recommendation algorithm to provide the user with a most appropriate service-related information. The flow of the public elderly service is shown in Figure 2.

### 2.2. Principles of Collaborative Filtering Recommendation Models and Their Drawbacks

Content-based recommendation means that other objects with similar attributes are used as recommendations based on the object selected by the user. Content-based recommendations do not need to be based on the user's evaluation opinion of the object. For example, in the field of elderly care services, when an elderly person often chooses a certain type of service, the elderly care recommendation system will recommend more similar services (from different providers) to them. Content-based recommendation algorithms need to extract the content features of the recommended objects, then compare these features with the user's needs, and recommend the goods with a better match [28–30].

The key problem in content-based recommendation is to calculate the degree of correlation between the relevant features of the recommended object and the user's interests.

(1) $$\begin{array}{c} u\left(c,s\right)=score\left(userprofile,content\right)\text{,} \end{array}$$

where *u*(*c*, *s*) is the recommendation degree of the recommendation object *S* for user c. userprofile is the interests of the user, and *content* is the content feature of the recommendation object model. There are many ways to calculate score, and the most commonly used one is the distance between cosine and included angle of vector.

(2) $$\begin{array}{c} u\left(c,s\right)=\cos\;\left(w_{c},w_{s}\right)=\frac{\overset{1}{\underset{i=1}{\sum }}w_{i,c}w_{i,s}}{\sqrt{\overset{r}{\underset{i=1}{\sum }}w_{i,c}^{2}\sqrt{\overset{r}{\underset{i=1}{\sum }}w_{i,s}^{2}}}}, \end{array}$$

where *w*_*c*_ is a vector representation of the user's interests, *w*_*s*_ is a vector representation of the object's content characteristics, *w*_*i*,*c*_ is the weight of keyword *i* with respect to the recommended object *c*, and *w*_*i*,*s*_ is the weight of keyword *i* with respect to user *s*. Finally, the top-ranked object is calculated as the recommendation result and fed back to the user. This is a content-based recommendation strategy.

The disadvantage of content-based recommendation strategies lies in the content extraction of the recommended objects [31–33]. At present, content recommendation based on text mining and text matching is a relatively mature approach. However, along with the emergence of a large number of media resources, many sounds, videos, and images cannot get to achieve effective content feature extraction. Therefore, content-based recommendation strategies are not applicable in this category of web resources.

**Figure 3:**
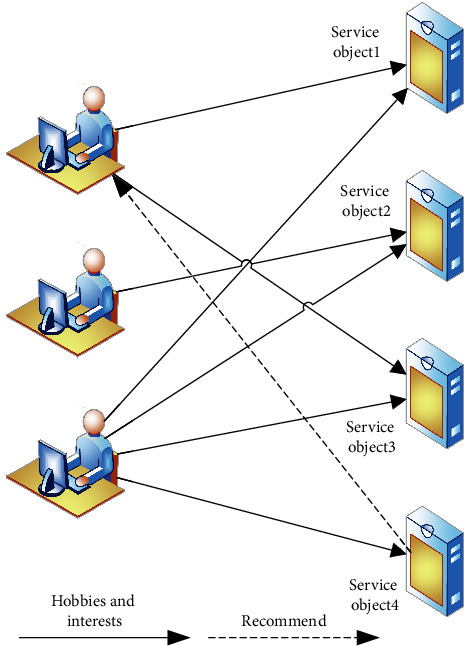
Recommendation process based on collaborative filtering.

Collaborative filtering recommendations are a relatively widely used recommendation algorithm today. Collaborative filtering recommendations are made through scoring prediction so that any form of resource content can be recommended to the user. The main idea of collaborative filtering recommendations is that “a user may prefer information resources chosen by other users with similar preferences.” In other words, the collaborative filtering recommendation algorithm needs to find objects that are similar to the preferences of an elderly user in order to make recommendations. The core operation of the strategy is to find similar users (nearest neighbours). The recommendation process based on collaborative filtering is shown in Figure 3.

**Figure 4:**
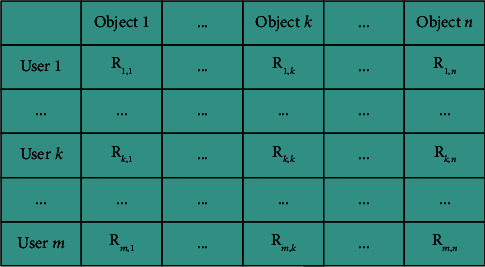
Evaluation matrix between elderly users and objects.

According to the above description, the collaborative filtering recommendation algorithm has two main steps [34], one is to find the nearest neighbours, and the other is to generate a recommendation list. After the nearest neighbours are found, then user similarity comparison is performed. The higher the similarity of the users, the more similar their preferences are, thus enabling the recommendation process. The similarity between an elderly user *u* and an elderly user *vis* denoted by sim(*u*, *v*). The elderly users' evaluation of the recommended object can be represented by an m-dimensional vector. Therefore, calculating the similarity between elderly users is to calculate the similarity between different m-dimensional vectors.  Figure 4 shows the evaluation matrix between the elderly users and the objects. Different columns can represent the similarity between objects, and different rows can represent the similarity between elderly users.

There are some drawbacks to collaborative filtering recommendation algorithms when applied to public elderly care services. For example, when a new user enters, the model does not have access to information about their daily preferences and therefore cannot make recommendations. The cold start problem occurs when new users have no information about their evaluation categories, which is a difficult area of research in recommender systems. Also when the size of the data increases, the evaluation gap between elderly users will increases, creating a sparsity problem. These are some of the shortcomings of collaborative filtering recommendation algorithms.

Both of these algorithms have their own strengths and weaknesses. There are some unavoidable problems that arise when using one of them alone. Currently, in practical applications, many recommendation algorithms are combined to obtain a better recommendation process. Therefore, this paper proposes to combine the XGBoost algorithm and the collaborative filtering algorithm to solve the recommendation cold start problem and the sparsity problem for elderly users.

## 3. The Proposed Hybrid Collaborative Filtering Algorithm

### 3.1. Basic Idea of the Proposed Algorithm

In order to improve the effectiveness and accuracy of the collaborative filtering results, this paper assigns weights to the scoring values of the traditional collaborative filtering algorithm and the scoring values of the XGBoost recommendation algorithm. The level of the new scoring results is used to achieve personalised recommendations for elderly users in the public elderly service system. In the content-based recommendation algorithm, the XGBoost model solves the problems of memory limitation in the similarity calculation, thus improving the execution efficiency.

First, a list of elderly user and item is created. In this list, the number of visits to certain item *i* by elderly user *u* can be mined, so that the elderly user's preferences can be understood based on the number of visits. In the public pension service system, when two elderly users ask the same item *i* more than three times, the two elderly users are determined to be the nearest neighbours. In the public elderly service system, all items are classified according to their similarity.

According to the average score of item *I* in the nearest neighbour, the recommendation system can derive the predicted scorings of user *u* for item *i*, thus solving the cold start problem for recommendations. If user *u*'s nearest neighbours have not had any scorings for item *i*, the user is removed from the nearest neighbours.

(3) $$\begin{array}{c} P_{u,i}=\frac{1}{k}\underset{v\in N_{u}\cap S\left(i\right)}{\sum }M_{v,i}\text{,}\\ P_{u,i}=\frac{\underset{v\in N_{u}\cap S\left(i\right)}{\sum }sim\left(u,v\right)M_{v,i}}{\underset{v\in N_{u}\cap S\left(i\right)}{\sum }\left|sim\left(u,v\right)\right|}\text{,} \end{array}$$

where sim(*u*, *v*) denotes the similarity which can be obtained by the similarity calculation method.

(4) $$\begin{array}{c} P_{u,i}=\bar{M_{u}}\cdot \frac{\underset{v\in N_{u}\cap S\left(i\right)}{\sum }sim\left(u,v\right)\cdot \left(M_{v,i}-\bar{M_{v}}\right)}{\underset{v\in N_{u}\cap S\left(i\right)}{\sum }\left|sim\left(u,v\right)\right|}\text{,}\\ sim\left(i,j\right)=\frac{i\cdot j}{\left\|i\right\|\left\|j\right\|}\text{,} \end{array}$$

where $\bar{M_{u}}$ and $\bar{M_{v}}$ denote the mean of user *u*'*s* and *v*'s scorings, respectively.

(5) $$\begin{array}{c} \bar{M_{u}}=\frac{1}{N\left(I\left(u\right)\right)}\underset{i\in I_{u}}{\sum }M_{ui}\text{.} \end{array}$$

The top *N* items with higher predicted scores are recommended to the user. Let the collaborative filtering recommendation algorithm be *T*_1_, and the XGBoost recommendation algorithm be *T*_2_, and then, the scoring result of the hybrid recommendation algorithm *T is* calculated as follows:

(6) $$\begin{array}{c} T=aT_{1}+\left(1-a\right)T_{2}\text{.} \end{array}$$

When user *u* evaluates item *i, the weight* of the collaborative filtering model is *α*_ui_, and the weight of the XGBoost model is *β*_ui_.

(7) $$\begin{array}{c} P_{ui}=\alpha_{ui}M_{ui}+\beta_{ui}M_{ui}^{\prime }+e\text{,} \end{array}$$

where *e* indicates an interference item.

The data set consisting of *α*_ui_ and *β*_ui_ is a sparse set, so most of the data values are zero. *α*_ui_ and *β*_ui_ can be written as *α*_*u*_ and *β*_*u*_ during the fitting process.

(8) $$\begin{array}{c} P_{ui}=\alpha_{u}M_{ui}+\beta_{u}M_{ui}^{\prime }+e\text{,} \end{array}$$

where *α*_*u*_+*β*_*u*_=1.

Finally, we need to solve the highest scoring value for personalised recommendations for elderly users.

(9) $$\begin{array}{c} \min_{\alpha_{u},\beta_{u}}\;=\underset{R\in S}{\sum }\left(M_{u,j}-\bar{M_{u}}\right){}^{2}\text{.} \end{array}$$

**Figure 5:**
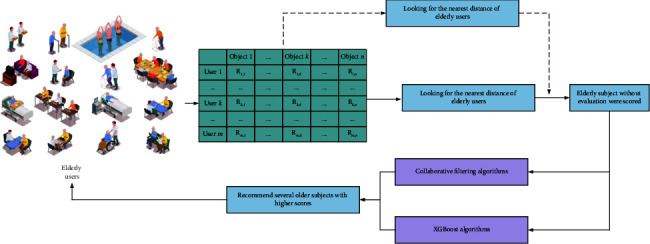
Hybrid collaborative filtering recommendation model for elderly users.

A collaborative filtering recommendation model for elderly users is an automatic behaviour. This model can make implicit judgements based on the behaviour of the elderly in order to get a reasonable recommendation solution. This model does not require any active behaviour from the user, such as filling out some questionnaires. In addition, the model can also handle unstructured and complex objects such as music, images, and videos. The hybrid collaborative filtering recommendation model for elderly users is shown in Figure 5.

### 3.2. Hybrid Similarity Calculation for Users

Similarity calculation for elderly users is a key part of the hybrid collaborative filtering recommendation model. In this paper, the hybrid similarity calculation is implemented by combining the similarity of elderly users' scorings with the similarity of elderly users' features. The similarity of elderly user scorings is calculated as follows:

(10) $$\begin{array}{c} {sim}_{p}\left(i,j\right)=\frac{\underset{p\in P}{\sum }\left(r_{i,p}-{\bar{r}}_{a}\right)\left(r_{j,p}-{\bar{r}}_{b}\right)}{\sqrt{\underset{p\in P}{\sum }{\left(r_{i,p}-{\bar{r}}_{a}\right)}^{2}}\sqrt{\underset{p\in P}{\sum }{\left(r_{j,p}-{\bar{r}}_{b}\right)}^{2}}}\text{,} \end{array}$$

where *p* is the scoring of items *p* by elderly user *i*, ${\bar{r}}_{a}$ is the average scoring of elderly user *a*, *r*_*j*,*p*_ is the scoring of items *p* by elderly users *j*, and ${\bar{r}}_{b}$ is the average scoring of elderly user *b*.

The similarity of characteristics of elderly users is calculated as follows:

(11) $$\begin{array}{c} {sim}_{r}\left(i,j\right)=\frac{\underset{p\in P}{\sum }c_{ip}\cap c_{jp}}{\underset{p\in P}{\sum }c_{ip}\cup c_{jp}}\text{,} \end{array}$$

where *c*_ip_ represents the *p*-th feature attribute of the elderly user *i*.

Mixed similarity is calculated as follows:

(12) $$\begin{array}{c} sim\left(i,j\right)=\alpha \cdot {sim}_{p}\left(i,j\right)+\left(1-\alpha \right)\cdot {sim}_{r}\left(i,j\right)\text{,} \end{array}$$

where*α* indicates the balance coefficient of the similarity measure for the two elderly users.

The traditional collaborative filtering recommendation algorithm scores according to the nearest neighbour similar to the user and takes the object with higher score as the recommendation result [35]. However, the proposed hybrid collaborative filtering recommendation algorithm builds on this by adding balancing coefficients corresponding to the characteristics of different elderly users, making the results of the hybrid similarity calculation more reflective of the attribute characteristics of the elderly users themselves. The proposed hybrid collaborative filtering recommendation algorithm more intuitively reflects the affinity relationship between users, so as to better realize the recommendation task of public pension services.

## 4. Experimental Results and Analysis

### 4.1. Experimental Environment and Setup

In order to verify the effectiveness of the proposed hybrid collaborative filtering recommendation algorithm, an example test was conducted using Matlab. The hardware and software environment related to the experiments are shown in Table 2. In addition, all the experiments involved in this study were conducted under the same hardware and software environment. The parameter settings for the hybrid collaborative filtering recommendation algorithm proposed during the experiments are shown in Table 3.

### 4.2. Experimental Data

The experimental data come from a public elderly service system with 1000 elderly users and contain 6000 relevant resource information. The maximum number of adjacent users in the sample set is 50, the total number of user scoring sets is 1000, and the total number of items is 10000. To verify the effectiveness of the hybrid algorithm, the performance of the proposed hybrid collaborative filtering was compared with XGBoost and collaborative filtering.

In this paper, different proportions of data from the above dataset are randomly selected as experimental samples to form 60%, 70%, 80%, and 90% sparsity test sets to verify the performance of the proposed algorithm under different sparsity. The sparsity is calculated as shown in the following equation:

(13) $$\begin{array}{c} \text{Sparsity}=1-\frac{F_{1}}{F_{2}\times F_{3}}\times 100\%\text{,} \end{array}$$

where *F*_1_ indicates the number of comments from users, *F*_2_ the number of users, and *F*_3_ the number of items.

A dictionary of feature attributes was also created for each elderly user in the dataset. The dictionary uses the hamming code to carry out the binary coding on the common attributes of the aged users, so as to facilitate the similarity calculation of the feature distances. At the same time, the attribute information in the feature dictionary was instantiated and randomly assigned to each elderly user in the test set.

### 4.3. Evaluation Indicators for Recommendation Systems

In this paper, we use three evaluation metrics commonly used in recommendation systems to measure the performance [36–38], namely, precision, mean absolute error (MAE), and recall. The smaller the MAE value is, the more accurate the prediction is. In addition, the sparsity of the user evaluation matrix is used as an experimental variable to compare the performance of the recommendations under different sparsity.

(14) $$\begin{array}{c} \text{Precision}=\frac{\left|S_{u}\cap S_{u}^{\prime }\right|}{\left|S_{u}\right|}\text{,}\\ \text{Recall}=\frac{\left|S_{u}\cap S_{u}^{\prime }\right|}{\left|S_{u}^{\prime }\right|}\text{,} \end{array}$$

where *S*_*u*_ indicates the number of resources preferred by the user in the set of recommended results, and *S*_*u*_′ indicates the number of all resources preferred by the user in the system.

(15) $$\begin{array}{c} MAE=\frac{1}{\left|count\left(S\right)\right|}\underset{R\in S}{\sum }\left|R_{u,j}-M_{u,j}\right|\text{,} \end{array}$$

where *R*_*u*,*j*_ denotes the predicted score of item *j* by user *u*, *M*_*u*,*j*_ denotes the actual score of item *j* by user *u*, and count(*S*) denotes the total number of user score in the set.

### 4.4. Recommended Performance Analysis

**Figure 6:**
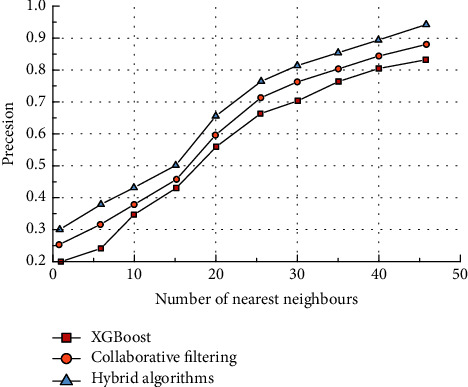
Comparison results for precision.

A total of two sets of experiments were conducted in this study. The first group performed the experimental procedure 15 times repeatedly at a sparsity of 60% and tallied the results for precision, MAE, and recall, respectively. The second group repeated the experimental procedure 20 times, corresponding to 4 different sparsity levels, in order to represent the effect of changes in sparsity on the upper three evaluation metrics. The comparative results of precision are shown in Figure 6.

It can be seen that the accuracy of all three recommendation algorithms gradually increases as the number of adjacent users increases. This indicates that all three recommendation algorithms can identify the set of items with high target user scorings in a large amount of data and obtain a high accuracy. Among the three algorithms, the accuracy of the recommendation algorithm based on the XGBoost model is relatively low, while the accuracy of the proposed algorithm is the highest. When the number of adjacent users reached 47, the recommendation accuracy of the proposed algorithm reached over 90%. The specific accuracy metrics are shown in Table 4.

**Figure 7:**
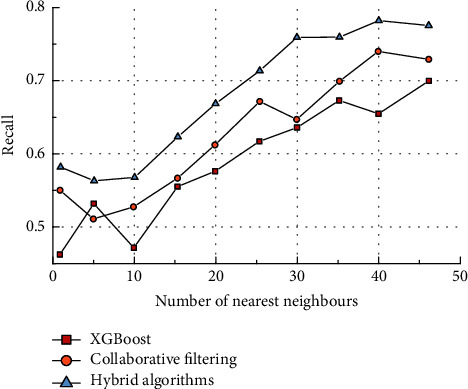
Comparison of recall rates.

It can be seen that the hybrid collaborative filtering algorithm achieves the highest accuracy rate of 94%. All three algorithms achieve good accuracy values when the number of neighbouring users is high enough. When the number of neighbouring users is small, all three recommendation algorithms have poorer accuracy rates. This is because the system cannot complete effective personalised recommendations when there is little data on a particular user's reviews in the Internet platform. When there are less data about the user's relevant characteristics, it is difficult to mine valuable data for that user and therefore cannot achieve effective recommendations based on user preferences and habits. The results of the recall comparison are shown in Figure 7.

**Figure 8:**
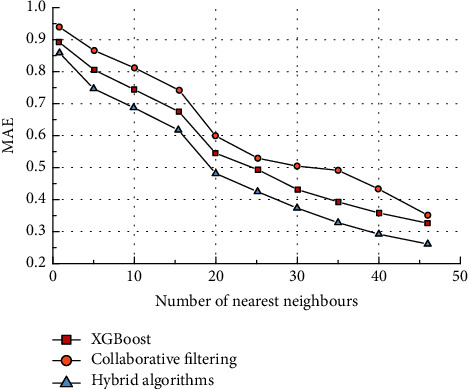
MAE comparison.

It can be seen that the recall rate gradually increases as the number of adjacent users increases. Compared to the other two algorithms, the hybrid algorithm has a better performance. The effective performance of the hybrid algorithm reaches over 75% when the number of neighbouring users reaches 45. As accuracy and recall are mutually constrained, the appropriate number of nearest neighbours should be selected during sample training in order to ensure the best balance between the two performances. In most cases, the collaborative filtering algorithm outperforms the XGBoost algorithm in terms of recall. However, when the number of neighbouring users is 5, the XGBoost algorithm outperforms the collaborative filtering algorithm. When the number of adjacent users was 30, both recall rates were the same, indicating that there was not much difference between the two algorithms in terms of recall rates. The MAE comparison results are shown in Figure 8.

It can be seen that as the number of nearest neighbours increases, the MAE gradually decreases (the predicted score values are closer to the actual score values). The MAE decreases rapidly when the number of nearest neighbours is in the range of 5–15. When the number of neighbouring users reaches 47, the MAE of the hybrid collaborative filtering algorithm drops to below 0.4.

**Figure 9:**
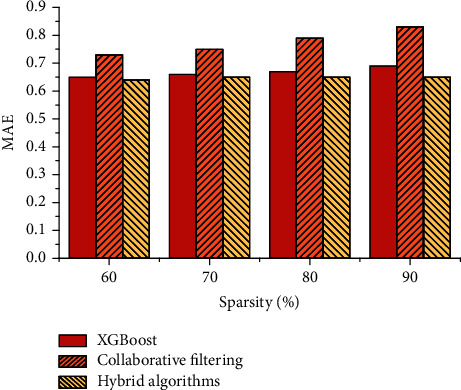
Effect of different sparsity on MAE.

As the sparsity changes, the MAE indicator changes as shown in Figure 9.

It can be found that as the sparsity rises, the error of the collaborative filtering algorithm becomes larger and larger, even exceeding 0.8, which indicates that the traditional collaborative filtering algorithm relies heavily on the number of user scorings. The hybrid collaborative filtering algorithm, however, can better suppress the problems caused by sparsity. The MAE of the XGBoost algorithm, on the other hand, remains largely unchanged.

**Figure 10:**
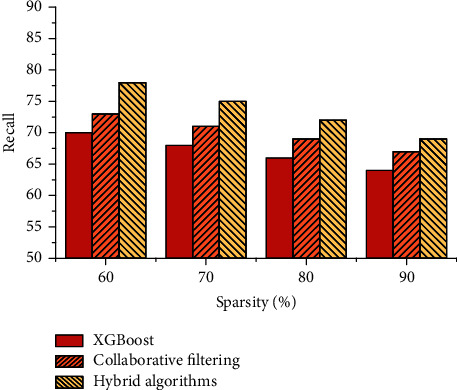
Effect of different sparsity on recall.

As the sparsity changes, the recall metric changes as shown in Figure 10.

It can be seen that the performance of the three recommended algorithms decreases when the sparsity increases, which is due to the relatively low number of evaluations by elderly users. The proposed hybrid collaborative filtering recommendation algorithm is more accurate and stable in recommending services to elderly users at the same sparsity.

## 5. Conclusion

In this paper, an intelligent recommendation method based on hybrid collaborative filtering is proposed and applied to a public elderly service system to complete personalised service recommendations in the public elderly service system. The XGBoost model and the collaborative filtering model are combined, thus solving the cold start problem and sparsity problem of recommendation for elderly users. The optimal objective function is solved by weighting parameters, thus solving the recommendation of elderly services for a special group of people. The experimental results show that the proposed hybrid collaborative filtering recommendation algorithm can more accurately recommend services to elderly users, and the results are more stable. The performance of the proposed algorithm with different weighting parameters will be further investigated in order to explore the optimal weight setting conditions.

## Figures and Tables

**Table 1:** Description of specific service details.

Service name	Service details
Daily care	Home delivery services for the elderly such as meal delivery, shopping for the elderly, and housekeeping services
Medical care	To provide disease prevention and treatment, health education, rehabilitation care, and mental health services for the elderly
Legal services	Provide legal advice, legal aid, and judicial mediation services for the elderly
Cultural education	Provides training, lectures, painting, chess, and recreation for the elderly
Sports & fitness	Fitness-related services for elderly people
Voluntary services	Provide talk and community care services for elderly people

**Table 2:** Experiment-related software and hardware environment.

Specification	Parameters
Processor	Intel core i5
Memory	8 GB
Hard disk	500 GB
Operating systems	Windows 7 professional
Programming software	Matlab R2010b

**Table 3:** Parameter settings for the hybrid collaborative filtering recommendation algorithm.

Parameters	Numerical values
Collaborative filtering weight *α*_ui_	0.8
XGBoost recommended weight *β*_ui_	0.2
Balance factor *α*	0.3

**Table 4:** Accuracy statistics for the three algorithms.

Number of nearest neighbours	Precision		
	XGBoost	Collaborative filtering	Hybrid algorithms
2	0.2	0.25	0.30
7	0.24	0.31	0.38
12	0.37	0.39	0.44
17	0.44	0.46	0.51
22	0.58	0.62	0.68
27	0.67	0.72	0.77
32	0.71	0.77	0.82
37	0.77	0.81	0.86
42	0.81	0.85	0.90
47	0.83	0.88	0.94

## Data Availability

The experimental data used to support the findings of this study are available from the corresponding author upon request.
